# MSCs‑derived exosomes attenuate ischemia-reperfusion brain injury and inhibit microglia apoptosis might via exosomal miR-26a-5p mediated suppression of CDK6

**DOI:** 10.1186/s10020-021-00324-0

**Published:** 2021-07-02

**Authors:** Chang Cheng, Xiuying Chen, Yuhan Wang, Wenchao Cheng, Xuzheng Zuo, Weiju Tang, Wen Huang

**Affiliations:** Department of Neurology, Xinqiao Hospital, Third Military Medical University (Army Medical University), No. 188 Xinqiaozheng Street, Chongqing, 400038 People’s Republic of China

**Keywords:** Exosomes, Mesenchymal stromal cells, miR-26a-5p, CDK6, Ischemia–reperfusion injury

## Abstract

**Background:**

This study aimed to explore the role of mesenchymal stromal cells (MSCs)-derived exosomes (MSCs-Exo) in the cerebral ischemia–reperfusion (I/R) injury.

**Methods:**

Exosomes were isolated from MSCs of adult C57BL/6J mice by the gradient centrifugation method. The expression of miR-26a-5p and CDK6 in MSCs-Exo and mice brain tissues were evaluated by qRT-PCR and western blot. miR-26a-5p mimics and miR-NC were transfected into MSCs, and exosomes were isolated from the MSCs stably expressing miR-26a-5p. Then MSCs-Exo-miR-26a-5p mimics or MSCs-Exo-miR-NC was injected into mice through the tail vein, or added into medium to stimulate BV-2 cells. Cell viability was evaluated by CCK-8 assay. Cell apoptosis was detected by flow cytometry. The apoptosis in brain tissues was evaluated by TUNEL staining assay. Bioinformatics analysis and luciferase reporter assay were performed to determine the binding relationship between miR-26a-5p and CDK6.

**Results:**

miR-26a-5p was downregulated and CDK6 was upregulated in MSCs-Exo of MCAO-mice and OGD-induced MSCs. MSCs-Exo-miR-26a-5p mimics significantly reduced cell apoptosis of OGD-injured BV-2 cells. MSCs-Exo-miR-26a-5p mimics significantly reduced the infarct volume of MCAO-induced mice. Luciferase reporter assay revealed that CDK-6 was a target of miR-26a-5p. In addition, MSCs-Exo-miR-26a-5p mimics significantly decreased the expression of CDK6 in both OGD-induced BV-2 cells and the brain tissues of MCAO-treated mice.

**Conclusion:**

Our results indicated that MSCs‑Exo attenuated I/R injury in mice by inhibiting microglia apoptosis might via exosomal miR-26a-5p mediated suppression of CDK6. Our study shed light on the application of MSC-Exo as a potential therapeutic tool for cerebral I/R injury.

## Background

Ischemic stroke is becoming a major leading cause of disability and death worldwide (Barthels and Das 2020). Approximately 85% of all reported strokes are due to cerebral ischemia that always occurs when an embolus or thrombus blocks the major cerebral artery and then leads to cell death (Powers 2020). The primary treatments for ischemic stroke are recanalization therapies, which are proved to replenish nutrients and oxygen, and also remove toxic metabolites (Phipps and Cronin 2020). Hence, the identification of new therapies or the understanding of critical mediators participated in the progression of cerebral ischemic stroke is still urgent.

Exosomes are nano-scale messengers that carry bio-molecular cargo of RNA, DNA, and proteins, and exosomes secreted stem cells have been reported that can regulate various autocrine and paracrine functions to alter cell micro-environment, followed by the progression in various human diseases (Sharma 2018). The potential clinical values of exosomes can be attributed to their characteristics including their surface markers and molecular cargoes (Dorayappan et al. 2016) their ability to cross the brain–blood barrier (BBB) (Kalani et al. 2014), and their potential functions as the mediators of the regenerative responses (Xin 2012). Recently, stem cell therapy has becoming a promising therapeutic option for some neurological disorders including Stroke, Parkinson’s disease, Amyotrophic Lateral Sclerosis and Huntington’s disease (Yoo et al. 2013). Because the greater benefit on tissue regeneration and repair than the stem cells themselves, stem cell-derived exosomes that are responsible for their therapeutic benefits have begun to be noticed. One of the most popular stem cell-derived exosomes which are widely applied in neurological diseases researches are mesenchymal stromal cells-derived exosomes (MSCs-Exo) (Luarte et al. 2016; Marote et al. 2016). However, the research about MSCs-Exo in cerebral ischemia–reperfusion (I/R) injury is lack and attracted us to pay more attention on it.

MicroRNAs (miRNAs) are found in both intracellular and extracellular environments, and also detected in exosomes participated cell–cell communication (Zhang 2015). Increasing reports have revealed a large number of exosome-derived miRNAs in cerebral ischemic stroke. For example, exosomal miR-223 is significantly upregulated in acute ischemic stroke, and high level of exosomal miR-223 is closely associated with the occurrence of acute ischemic stroke and stroke severity (Chen 2017). M2 microglia-derived exosomes has been showed to protect the mouse brain against I/R injury via miR-124 (Song 2019a). Exosomes-derived miR-26a-5p, a newly identified exosomal miRNA, has been revealed to play important roles in human diseases. Human bone MSCs-derived exosomes overexpressing miRNA-26a-5p significantly alleviate osteoarthritis development through downregulating PTGS2 (Jin et al. 2020). Dysregulation of exosomal miRNAs such as miR-26a-5p may affect the regulatory pathways associated with clopidogrel-induced liver injury (Freitas 2017). In addition, one previous study demonstrated that miR-26a-5p was downregulated in myocardial I/R injury, and overexpression of miR-26a-5p effectively improved cell viability and inhibits cell apoptosis in cardiomyocytes upon I/R injury by inhibiting PTEN expression (Xing and Guo 2020). Although exosomal miR-26a-5p played crucial functions in various diseases including I/R injury of some organs, its function and molecular mechanism in cerebral I/R injury remain unclear.

CDK6 is known as a classic cell cycle kinase by forming complexes with D-type cyclins, which can phosphorylate the retinoblastoma protein (Rb) to regulate the transition from G1 to S phase (Tigan et al. 2016). Previous studies reported that CDK6 was closely involved in the biological processes including cell proliferation, apoptosis and the transition from G1 to S phase in cerebral ischemic stroke (Li 2018; Demyanenko and Uzdensky 2017). Interestingly, Huang et al. found that miR-26a-5p inhibited the growth of breast cancer cells by downregulating the expression of CDK6 (Huang 2019), suggesting that CDK6 might be a direct or indirect downstream gene of miR-26a-5p. Meanwhile, a miRNA profiling and bioinformatics prediction performed by Canturk et al. indicated that the CDK6 was predicted to be a target of miR-26a-5p, and the axis was significantly associated with the progression of bladder cancer (Canturk 2014), further indicating that CDK6 might be a direct target of miR-26a-5p.

In the present study, we explored the role of exosomal miR-26a-5p/CDK6 axis in cerebral I/R injury. Taken together, our study demonstrated that MSCs‑derived exosomes overexpressing miR-26a-5p effectively reduced cell apoptosis of OGD-treated microglia cell line BV-2 cells in vitro, and also attenuated MCAO-induced infarct volume in mice, suggesting that exosomal miR-26a-5p might be a potential therapeutic target for cerebral ischemic stroke.

## Methods

### Isolation of MSCs-derived exosomes

Mesenchymal stem cells (MSCs) were isolated from six adult C57BL/6J mice by the whole bone marrow adherence method as previously described (Maria 2016). 2 × 10^6^ MSCs were cultured in 100 mm dishes, MSCs were washed with PBS and kept in fetal bovine serum (FBS)-free L-DMEM medium (Gibco, CA, USA) for 48 h when cells reached nearly 80–90% confluence. Then the supernatant was collected and subject to sequential centrifugation to obtain the exosomes according to a previous study (Théry et al. 2006). The precipitated exosomes were stored at − 80 ℃.

### Characterization of MSCs-derived exosomes

For transmission electron microscopy (TEM), the isolated exosomes were fixed with 1% glutaraldehyde, then a drop of fixed exosomes was spotted onto a formvar/carbon-coated grid and negatively stained with 3% aqueous phosphotungstic acid for 1 min. Subsequently, MSCs-derived exosomes were observed under TEM (Hitachi, Tokyo, Japan, SU-8010). For flow cytometry analysis of exosomes, MSCs-derived exosomes were mixed with 3 μm aldehyde/sulfate latex beads (Invitrogen, Batch Num: 979383) for 10 min with continuous rotation. 1 M glycine in PBS containing 2% BSA was added into the mixture to stop the reaction. Beads coated with exosomes were incubated with antibodies CD63-FITC (Lot: GR320523-9, Abcam), CD81-PE (Cat: MA5-17941, Invitrogen) at 37 ℃ for 25 min. Then a flow cytometry (FCM) (BD FACSCalibur) was used to detect the mesenchymal markers. In addition, the detection of exosomal markers was introduced in western blot assay by using corresponding antibodies including CD9, CD63, CD81, HSP70 and Calnexin.

### Animal model of cerebral ischemia–reperfusion (I/R)

A total of 30 adult C57BL/6J mice (8 weeks old and 250 g in weight) were provided from Anima Center of Third Military Medical University (Chongqing, China). All animal producers were approved by the Institutional Animal Care and Use Committee of Anima Center of Third Military Medical University (Chongqing, China). Mice with cerebral ischemia was induced by middle cerebral artery occlusion (MCAO) as described previously with minor modifications (Yang 1994). Reperfusion was performed by withdrawing the suture 1 h after MCAO. For sham group, mice received the same operation except MCAO procedure. 2 h after reperfusion, 200 μL/mice MSCs-exosomes-miR-NC or MSCs-exosomes-miR-26a-5p mimics (RiboBio, Guangzhou, China) were immediately injected through the tail vein. The mice in the control group were given an equal volume of normal saline (n = 6 in each group). All mice were divided into four groups: Sham group, MCAO group, MCAO + MSCs-exosome-miR-NC, and MCAO + MSCs-exosome-miR-26a-5p mimics group.

### Determination of infarct size

After intraperitoneal injection of 3% sodium pentobarbital, the brains of mice in different groups were removed, placed in 4% PFA overnight and then fully dehydrated in 30% sucrose for 2 days. The brain tissues were then cut into coronal sections with approximately 2 mm in thickness, and then quickly incubated in 2% TTC solution (Sigma) at 37 °C for 15 min. Subsequently, the sections were fixed by 4% paraformaldehyde and photographed under a × 100 optical microscope (Olympus Corporation). The image analysis software Image J 1.43 (National Institutes of Health) was used to evaluate the relative infarct percentage according to the following equation: infarct size = 100% × (infarcted volume/total brain volume).

### MSCs of OGD model

MSCs were cultured with in Dulbecco’s modified Eagle’s medium (DMEM, Gibco, USA) supplemented with 10% (v/v) fetal bovine serum (FBS, Biological Industries, USA), 100 U/mL penicillin and 100 μg/mL streptomycin (Gibco, USA) at 37 °C under 5% CO_2_. To mimics the ischemia condition in vitro, cells were firstly induced by oxygen–glucose deprivation (OGD) using deoxygenated glucose-free DMEM medium in an incubator with 95% N_2_, 5% CO^2^ for 1, 2 and 4 h, then cells were transferred into normal condition for an additional 24 h for re-oxygenation. Cells treated without OGD were used as the control group.

### Cell transfection

MSCs were transfected with 50 nM miR-26a-5p mimics or miR-NC (RiboBio, Guangzhou, China) by using Lipofectamine® 2000 Transfection Reagent (Gibco Life Technologies) according to the manufacturer’s instructions. The sequences used in this study as follows: miR-26a-5p mimics: 5′-UUCAAGUAAUCCAGGAUAGGCU-3′; miR-NC: 5′-UUCUCCGAACGUGUCACGUTT-3′. When needed, the exosomes were isolated from MSCs transfected with miR-26a-5p mimics or miR-NC and used for the treatment of microglia cell line BV-2 cells.

### The treatment of microglia

To simulate an in vivo environment of MCAO in microglia, BV-2 cells (Bioleaf, Shanghai, China) were induced by OGD/R treatment as similarly as the MSCs of OGD model above, and BV-2 cells received 2 h of OGD followed by 24 h of re-oxygenation. Then 200 μg/mL MSCs (transfected with miR-26a-5p mimics or miR-NC)-derived exosomes were added into the culture medium to explore the effect of MSCs-Exo on microglia function.

### CCK-8 assay

Cell viability was evaluated by using a Cell Counting Kit-8 (CCK-8, Dojindo Molecular Technologies, Gaithersburg, MD). In brief, MSCs were seeded into 96-well plates overnight. After the treatment, 10 μL of CCK-8 reagent was added to each well at 24, 48, 72 and 96 h and then incubated for another 4 h. The absorbance at 450 nm was detected with a microplate reader.

### Luciferase reporter assay

The putative binding sites between miR-26a-5p and 3′-UTR of CDK6 were predicted by starBase (http://starbase.sysu.edu.cn/). The mutant-type (MUT) of 3′-UTR (CDK6- MUT) and wild type (WT) of 3′-UTR (CDK6-WT) were amplified and cloned into the pmirGLO dual luciferase reporter vector (Promega, Madison, WI, USA). Then the luciferase reporter plasmids were co-transfected with miR-26a-5p mimics or miR-NC into 293 T cells by using Lipofectamine® 2000 Transfection Reagent. 48 h after transfection, cells were lysed and the relative luciferase activity was detected by the dual-luciferase reporter gene assay (Promega).

### RNA extraction and qRT-PCR analysis

Total RNA was extracted from cultured cells or mice brains by using Trizol reagent (Invitrogen). Single-strand cDNA was synthesized using a universal cDNA synthesis kit (Qiagen, Hilden, Germany) according to the manufacturer’s instructions. The expression of targets was tested with a fast real-time PCR system (7900 HT, ABI, Foster City, CA) by using a SYBR Green master mix (Qiagen). The relative expression change of targets was analyzed by the 2^−ΔΔCt^ method with GAPDH and U6 as the internal references. The primers used as follows: miR-26a-5p: forward: 5′-GACGGTACCTTGTCCCTGAATGTAACTCG-3′ reverse: 5′-GTTCTCGAGAAAGCAGTCCCAGCCTAAA-3′; U6: forward: 5′-CTCGCTTCGGCAGCACA-3′, reverse: 5′-AACGCTTCACGAATTTGCGT-3′; GAPDH forward: 5′-CAAGGTCATCCATGACAACTTTG-3′, reverse: 5′-GTCCACCACCCTGTTGCTGTAG-3′.

### Western blot

Total protein of mice brains or cultured cells was isolated by using RIPA lysis buffer (Beyotime Institute of Biotechnology). Approximately equal amounts of protein were separated by 10% SDS-PAGE and transferred onto PVDF membranes (Millipore). After blocking with 5% skim milk, the membranes were incubated with primary antibodies including CD9, CD63, CD81, HSP70, CDK6, Calnexin, cleaved Caspase3 (c-Caspase3), cleaved PARP, and GAPDH antibodies (diluted into 1:1000; Cell Signaling Technology, USA) overnight at 4 °C. On the next day, the membranes were incubated with HRP-labeled secondary antibody at room temperature for 1 h. After washing with TBS-T, the protein bands were visualized by ECL reagent and relative integrated density values were calculated using Image J software, with GAPDH as the internal reference. was quantified by Image-Pro Plus 6.0 software (Media Cybernetic).

### Apoptosis analysis

Cell apoptosis was analyzed by using a commercialized Annexin V-FITC/PI Apoptosis Detection Kit (YEASEN, Shanghai) according to the manufacturer’s protocols. Briefly, BV-2 cells were washed with PBS, and re-suspended in 100 μL of binding buffer, and 5 μL of Annexin V-FITC and 10 μL of propidium iodide (PI) were gently mixed and added into cell suspension, then incubated in the dark for 15 min. Finally, cell apoptosis was detected by flow cytometry (BD FACS Canto II, USA).

### In situ detection of fragmented DNA (TUNEL assay)

Brains tissues were collected and the apoptosis in vivo was evaluated TUNEL staining kit (YEASEN, Shanghai) as the manufacturer’s instructions. TUNEL positive brain cells were counted under a fluorescence microscope. Specially, cell nucleus dyed green were considered to be apoptotic cells, and the rate of apoptosis (%) was calculated as the percentage of TUNEL positive cell nucleus in 5 random fields for each sample.

### Statistical analysis

All data were presented as mean ± SD, and each experiment was repeated three times. Statistical analysis was performed by using GraphPad Prism 6.0 software. Groups comparison was performed with two tailed Student’s t test (two groups) or one-way analysis of variance followed by a post-hoc test (ANOVA; multiple groups). P < 0.05 was considered to be significant.

## Results

### Characterization of MSCs‑derived exosomes

**Fig. 1:**
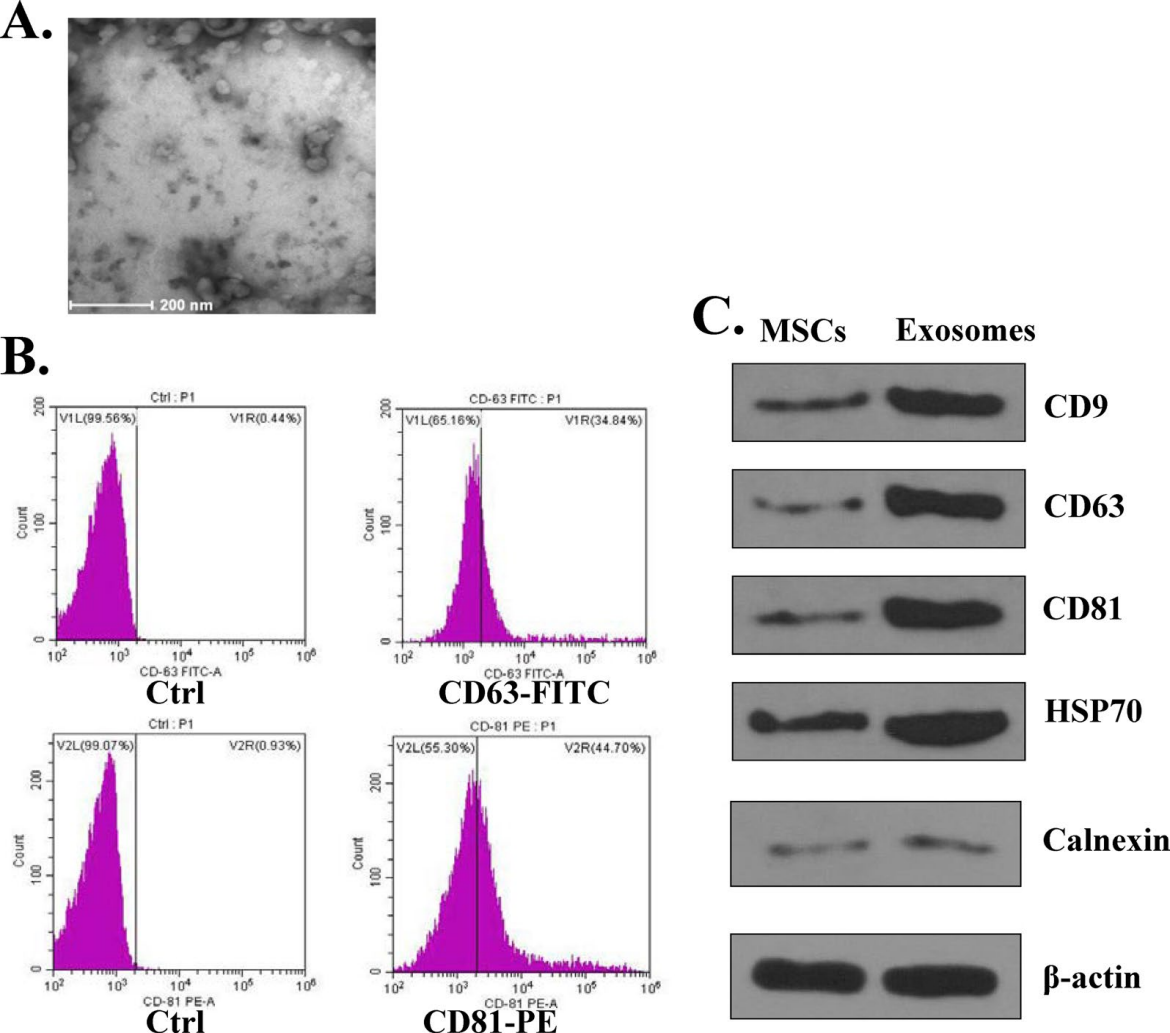
Characterization of MSCs‑derived exosomes. **A** Morphological change of MSCs-derived exosomes by TEM. **B** FACS analysis of exosome surface markers CD63 and CD81 in MSCs-derived exosomes. **C** The protein expression of exosome surface markers (CD9, CD63, CD81, HSP70, Calnexin) in MSCs and MSCs-derived exosomes was evaluated by western blot

To ensure the reliability of subsequent experiments, we firstly detected the characterization of MSCs‑derived exosomes. Under the transmission electronic microscope (TEM), MSCs-derived exosomes showed widespread, derangement distribution, and conglobation in some areas, and the vesicles exhibited typical exosome morphology with a dimension varying from 30 to 150 nm (Fig. 1A). Flow cytometry assay indicated that CD63 and CD81 positive rates were 34.84% and 44.70%, respectively (Fig. 1B), suggesting that MSCs-exosomes isolated were of certain purity. Meanwhile, the expression of exosomal markers was evaluated by western blot, and the results showed that the exosomal markers including CD9, CD63, CD81 and HSP70 were all expressed in MSCs and MSCs-derived exosomes (Fig. 1C). In addition, there was obvious change on the expression of Calnexin in MSCs and MSCs-derived exosomes (Fig. 1C). These results suggested that the isolated exosomes could be used for the subsequent experiments.

### miR-26a-5p was downregulated and CDK6 was upregulated in MSCs-derived exosomes of MCAO and OGD model

**Fig. 2:**
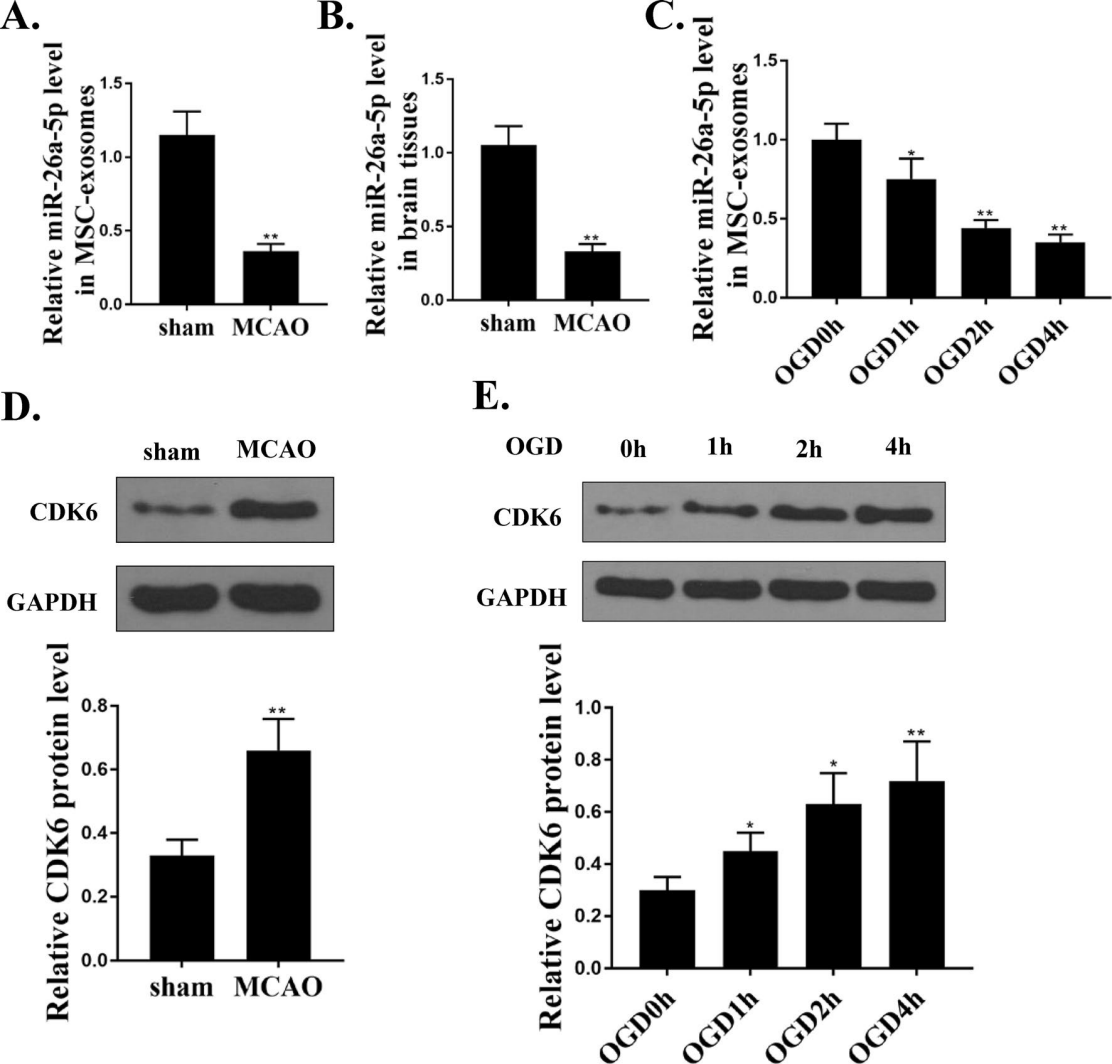
MiR-26a-5p was downregulated and CDK6 was upregulated in MSCs-derived exosomes of MCAO and OGD model. **A** and **B** The mRNA expression of miR-26a-5p in MSCs-derived exosomes (**A**) and brain tissues (**B**) of MCAO-induced mice was evaluated by qRT-PCR. **C** The level of miR-26a-5p in exosomes of OGD-treated MSCs at indicated time was evaluated by qRT-PCR. **D** The protein expression of CDK6 in MSCs-derived exosomes of MCAO-induced mice was evaluated by western blot. **E** The protein expression of CDK6 in exosomes of OGD-treated MSCs at indicated time was evaluated by western blot. *p < 0.05, **p < 0.01

Next, we explored the expression of miR-26a-5p and CDK6 in exosomes of cerebral I/R model in vitro and in vivo. Compared with sham group, the expression of miR-26a-5p was significantly downregulated both in MSCs-derived exosomes (p < 0.01, Fig. 2A) and brains tissues (p < 0.01, Fig. 2B) of MCAO-induced mice. Similarly, the level of miR-26a-5p in the exosomes of OGD-induced MSCs was also downregulated compared with that in control group (without OGD treatment) (p < 0.05, Fig. 2C). In addition, the protein level of CDK6 was increased in MSCs-derived exosomes of MCAO-induced mice compared with that in sham group (p < 0.01, Fig. 2D). Meanwhile, the expression of CDK6 was also upregulated in the exosomes of OGD-induced MSCs compared with that in control group (p < 0.05, Fig. 2E). These results revealed that miR-26a-5p was downregulated and CDK6 was upregulated in MSCs-derived exosomes of MCAO and OGD model.

### miR-26a-5p mimics reversed the effects of MSCs-derived exosomes in reducing cell apoptosis of OGD-injured microglia

**Fig. 3:**
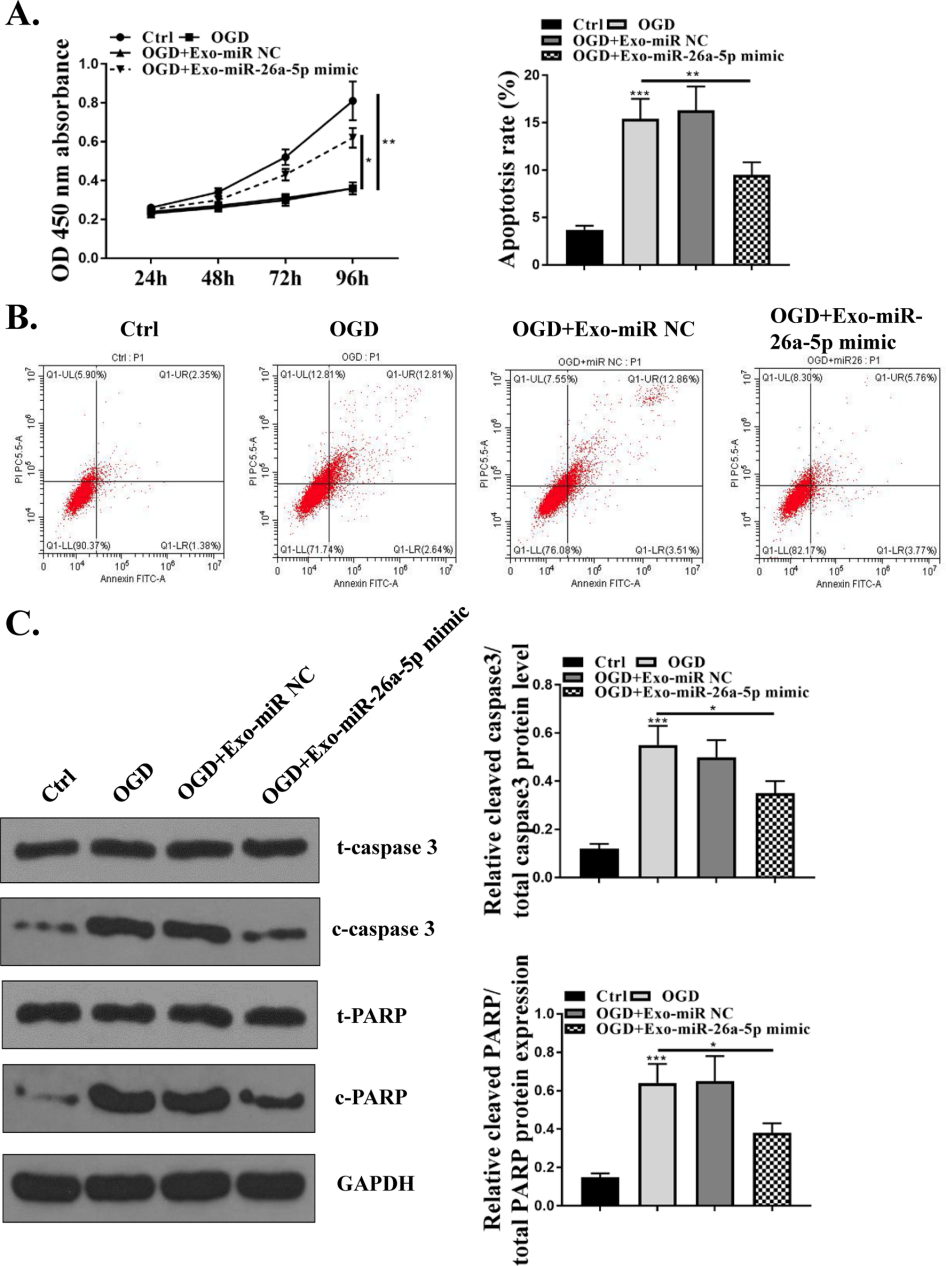
miR-26a-5p reversed the effects of MSCs-derived exosomes in reducing cell apoptosis of OGD-injured microglia. BV-2 cells were treated by OGD for the given time, and then treated with 200 μg/mL Exo-miR‐26a-5p mimics or Exo-miR-NC. **A** Cell viability was evaluated by CCK-8 assay. **B** Cell apoptosis was detected by flow cytometry. **C** The protein expression of apoptosis-related makers (cleaved caspase-3, total caspase-3, cleaved RARP and total RARP) was evaluated by western blot. *p < 0.05, **p < 0.01, ***p < 0.001

To further explore the effect of exosomal miR-26a-5p on microglia function, BV-2 cells were treated by OGD for the given time, and then treated with 200 μg/mL MSCs-Exo-miR‐26a-5p mimics or MSCs-Exo-miR-NC. CCK-8 assay indicated that OGD treatment significantly reduced cell viability compared with that in control group (p < 0.01), and OGD-treated BV-2 cells cultured with MSCs-Exo-miR‐26a-5p showed a greater cell viability than MSC-Exo-miR-NC group (p < 0.05, Fig. 3A). Meanwhile, OGD treatment significantly exacerbated the apoptosis rate of BV-2 cells compared with that in control group (p < 0.001), and the elevated apoptosis of BV-2 cells stimulated by OGD were obviously reduced by MSCs-Exos-miR-26a-5p mimics compared with MSCs-Exo-miR-NC group (p < 0.01) (Fig. 3B). In addition, the expression of apoptosis-related proteins was evaluated by western blot, and the results showed that the ratio of cleaved-caspase 3/total cleaved caspase-3 and cleaved-PARP/total PARP were increased in BV-2 cells after OGD treatment compared with that in control group (p < 0.001), and the effects were significantly reversed by MSCs-Exo-miR-26a-5p mimics compared with MSCs-Exo-miR-NC (p < 0.05) (Fig. 3C). These results indicated that overexpression of miR-26a-5p could reverse the effects of MSCs-derived exosomes in reducing cell apoptosis of OGD-injured microglia.

### MSCs-Exo-miR-26a-5p mimic attenuated ischemia–reperfusion injury in MCAO/R model

**Fig. 4:**
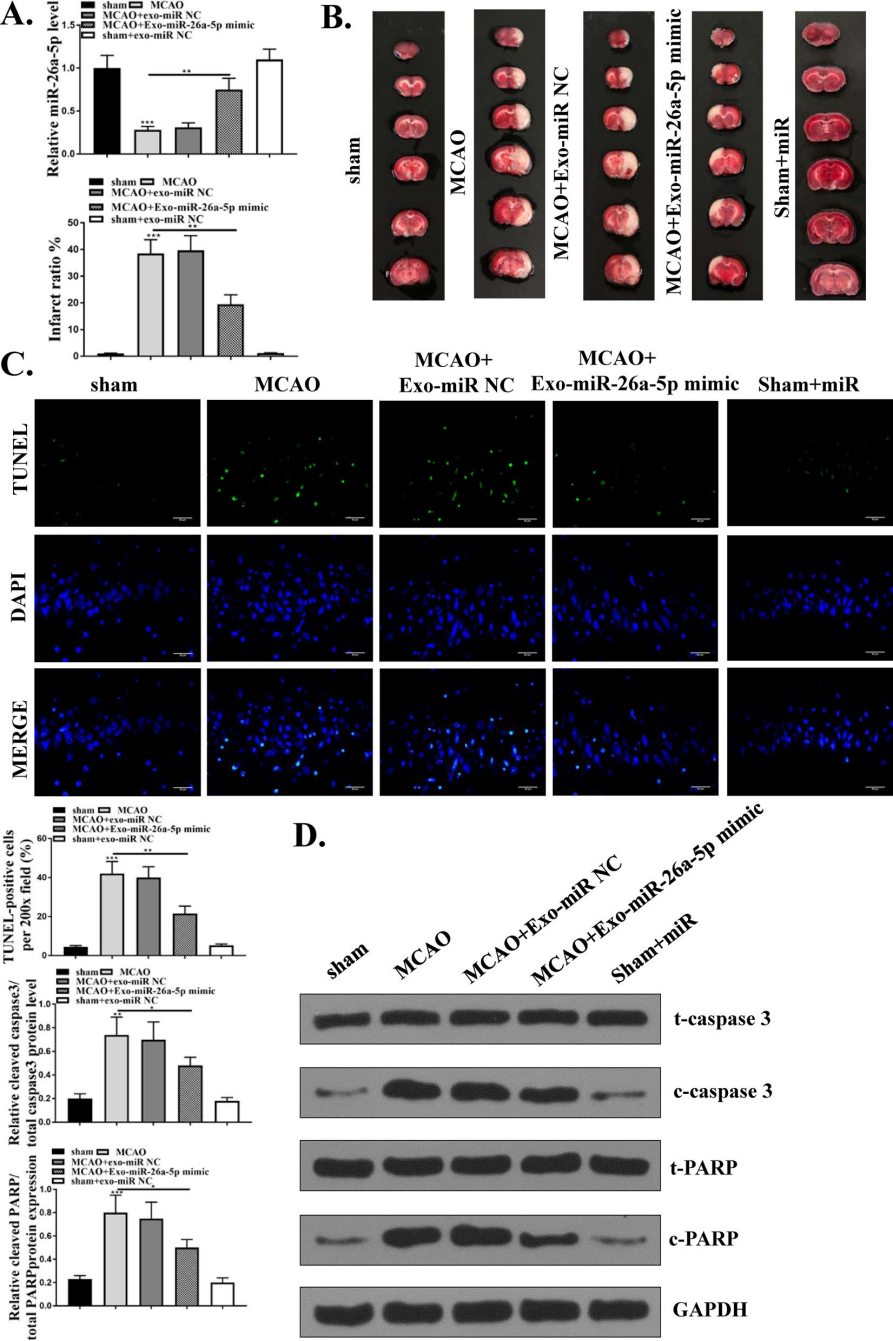
MSCs-Exo-miR-26a-5p mimic attenuated ischemia–reperfusion injury in MCAO/R model. Mice were treated with MCAO/R, and injected with 200 μL/mice MSCs-exosomes-miR-NC or MSCs-exosomes-miR-26a-5p mimics. **A** The mRNA level of miR-26a-5p in brain tissues was evaluated qRT-PCR. **B** Representative brain sections of MCAO/R induced mice were detected by TTC staining. **C** TUNEL staining in brain tissues. **D** The protein level of apoptosis-related markers (cleaved caspase-3, total caspase-3, cleaved RARP and total RARP) in brain tissues was detected by western blot. *p < 0.05, **p < 0.01, ***p < 0.001

We then explored the neuroprotective effect of MSCs-Exo-miR-26a-5p in vivo through then injection of 200 μL/mice MSCs-exosomes-miR-NC or MSCs-exosomes-miR-26a-5p mimics by the tail vein after MCAO/R treatment. The expression of miR-26a-5p in brain tissues was firstly detected by qRT-PCR and the results showed that MCAO/R treatment significantly reduced miR-26a-5p level compared with sham operation (p < 0.001), and compared with MSCs-Exo-miR-NC group, MSCs-Exo-miR-26a-5p mimics obviously attenuated MCAO/R-induced downregulation of miR-26a-5p (p < 0.01) (Fig. 4A). The results of TTC staining (Fig. 4B) showed that MCAO/R treatment significantly enhanced infarct volume compared with sham operation (p < 0.001), and compared with Exo-miR-NC group, Exo-miR-26a-5p mimics markedly reduced the infarct percentage (p < 0.01). Meanwhile, cell apoptosis in brain tissues was determined by TUNEL staining and the results indicated that TUNEL positive cells were increased after MCAO/R treatment compared with sham operation (p < 0.001), and Exo-miR-26a-5p mimics obviously reduced TUNEL positive cells of mice brains after MCAO/R treatment compared with Exo-miR-NC group (p < 0.01) (Fig. 4C). In addition, the expression of apoptosis-related proteins in brain tissues was also evaluated by western blot and the results showed that MCAO/R-treatment significantly increased the ratio of cleaved-caspase 3/total cleaved caspase-3 and cleaved-PARP/total PARP in brain tissues compared with sham operation (p < 0.01), Exo-miR-26a-5p mimics obviously reduced the ratio of cleaved-caspase 3/total cleaved caspase-3 and cleaved-PARP/total PARP in mice brains induced by MCAO/R (p < 0.05, Fig. 4D). In addition, Exo-miR-26a-5p mimics exhibited no obvious effect on miR-26a-5p expression, infarct volume, the ration of cleaved-caspase 3/total cleaved caspase-3 and cleaved-PARP/total PARP in sham-operated mice (Fig. 4A–C). These results suggested that MSCs-Exo-miR-26a-5p mimics attenuated ischemia–reperfusion injury in MCAO/R model in vivo.

### The effect of miR-26a-5p was partially mediated by CDK6

**Fig. 5:**
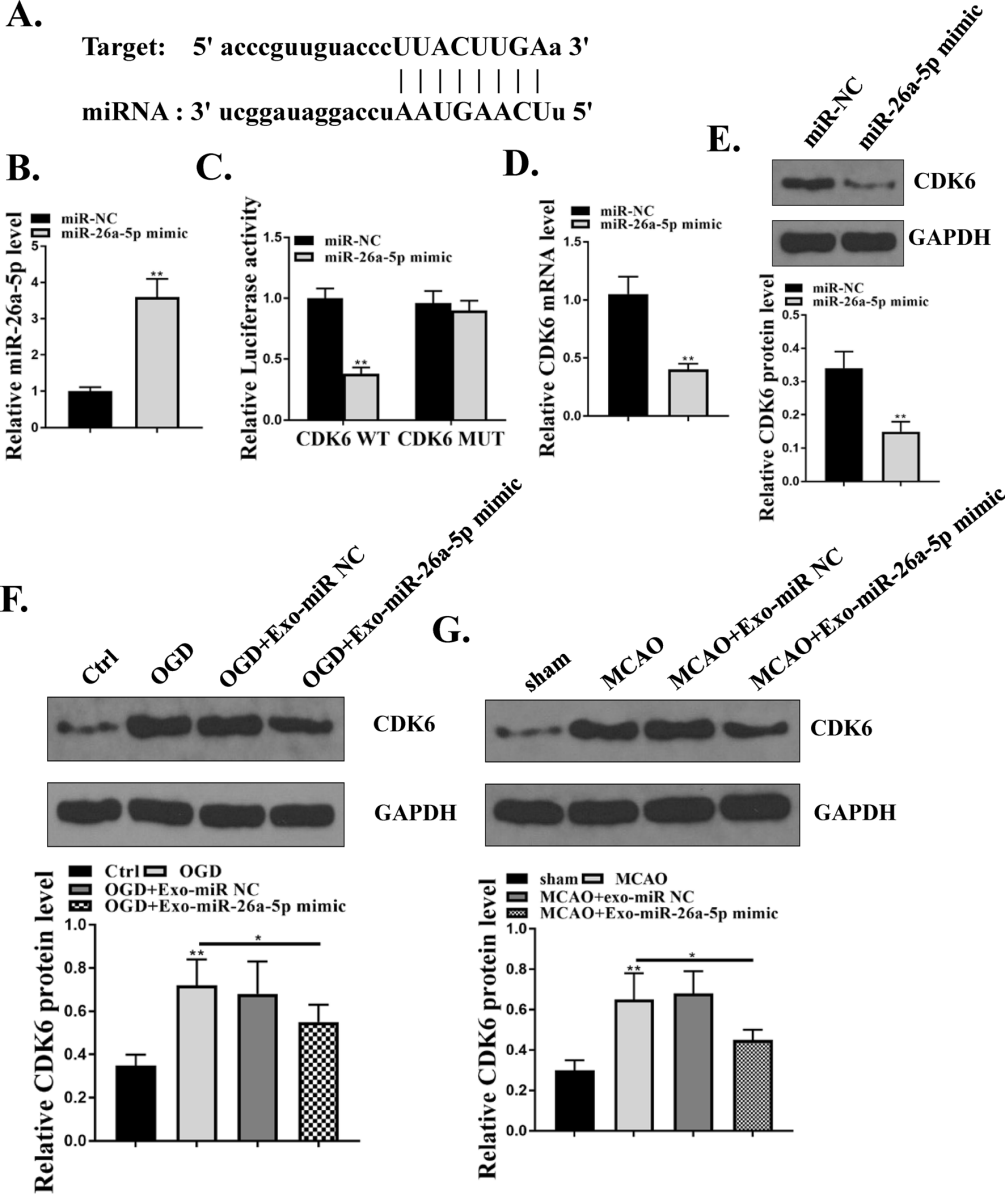
The effect of miR-26a-5p was partially mediated by CDK6. **A** The putative binding site between miR-26a-5p and CDK6 was predicted by Targetscan. **B** 293 T cells were transfected with miR-26a-5p mimics or miR-NC, and the mRNA level of miR-26a-5p was detected by qRT-PCR. **C** 293 T cells were co-transfected with miR-26a-5p mimics or miR-NC and Luc-CDK6-WT or Luc-CDK6-MUT, and the relative luciferase reporter activity was evaluated by dual luciferase reporter system. **D** and **E** BV-2 cells were transfected with miR-26a-5p or miR-NC, and the expression of CDK6 was detected by qRT-PCR (**D**) and western blot (**E**). **F** BV-2 cells were treated by OGD/R, and then treated with 200 μg/mL Exo-miR-26a-5p mimics or Exo-miR-NC, and the protein level of CDK6 was evaluated by western blot. **G** Mice were induced by MCAO/R, and then injected with 200 μL/mice Exo-miR-NC or Exo-miR-26a-5p mimics. The protein level of CDK6 in brain tissues was detected by western blot. *p < 0.05 **p < 0.01

To further determine the molecular mechanism of miR-26a-5p, we analyzed the sequence of the 3′-UTR of the human CDK6 gene. We found that there was a putative binding site between miR-26a-5p and 3′-UTR of CDK6 by using Targetscan software (http://www.targetscan.org), suggesting that CDK6 might be a target of mIR-26a-5p (Fig. 5A). Then miR-26a-5p mimics or miR-NC was transfected into 293 T cells and qRT-PCR showed that miR-26a-5p mimics significantly increased the miR-26a-5p level compared with miR-NC (p < 0.01, Fig. 5B). Meanwhile, luciferase reporter assay was performed in 293 T cells and the results indicated that miR-26a-5p mimics obviously reduced the relative luciferase activity of CDK6-WT compared with miR-NC (p < 0.01), but had no change on CDK6-MUT (Fig. 5C). Next, miR-26a-5p mimics or miR-NC was transfected into BV-2 cells and we found that miR-26a-5p mimics significantly decreased the expression of CDK6 at both mRNA level (p < 0.01) and protein level (p < 0.01) compared with miR-NC (Fig. 5D and E). Moreover, we found that OGD/R treatment significantly increased CDK6 level in BV-2 cells compared with control group (p < 0.01), and Exo-miR-26a-5p mimics obviously reduced CDK6 level compared with Exo-miR-NC group (p < 0.05) (Fig. 5F). In addition, MCAO/R treatment significantly increased CDK6 level in brain tissues of MCAO/R treated mice compared with that in sham operation (p < 0.01), and Exo-miR-26a-5p mimics markedly reduced CDK6 level in mice brains induced by MCAO/R treatment compared with Exo-miR-NC group (p < 0.05) (Fig. 5G). These data suggested that MSCs-derived exosomes overexpressing miR-26a-5p attenuated cerebral I/R injury might through targeting CDK6.

## Discussion

In this study, we explored the role of exosomal miR-26a-5p in cerebral I/R injury, and found that miR-26a-5p was downregulated in MSCs-derived exosomes of MCAO and OGD model. In addition, 50, 100 and 200 μL MSCs-Exo-miR-26a-5p were used, and we found that there was no obvious effect on cell viability, apoptosis and infarct volume when 50 and 100 MSCs-Exo-miR-26a-5p were applied. And, 200 μL MSCs-Exo-miR-26a-5p mimics effectively reduced cell apoptosis of BV-2 cells submitted to OGD/R treatment, and also reduced infarct volume of mice submitted to MCAO/R treatment by elevating the expression of CDK6. Our results provided that MSCs‑Exo overexpressing miR-26a-5p could effectively attenuate I/R brain injury through targeting CDK6, suggesting that miR-26a-5p might be a novel therapeutic strategy.

A series of miRNAs have been found to be loaded by exosomes in different types of cells, and play crucial roles in neuron injury (Deng 2019). For example, miRNA-181a overexpression in MSCs-derived exosomes suppresses inflammatory response after myocardial I/R injury (Wei 2019). Cortical neuron-derived exosomal miRNA-181c-3p inhibits neuro-inflammation by downregulating CXCL1 in astrocytes of a rat model with ischemic brain injury (Song 2019b). Exosomes derived from miR-138-5p-overexpressing bone marrow-derived mesenchymal stem cells confers neuroprotection to astrocytes following ischemic stroke via the inhibition of LCN2 (Deng 2019). Exosome-shuttled miR-92b-3p from ischemic preconditioned astrocytes protects neurons against oxygen and glucose deprivation (Xu 2019). Exosomal miR-26a-5p level is decreased in human umbilical cord derived mesenchymal stem cells (hUCMSCs), and exosomal miR-26b-5p from hUCMSCs represses M1 polarization of microglia by targeting CH25H to inactivate the TLR pathway, then finally relieves nerve injury after cerebral I/R (Li, 2020). In this study, we found that miR-26a-5p was downregulated in MSCs-derived exosomes of MCAO and OGD model. To explore the effect of exosomal miR-26a-5p in cerebral I/R injury, miR-26a-5p overexpressing exosomes were isolated from MSCs and used to stimulate BV-2 cells or injected into MCAO/R induced mice. MSCs-Exo-miR-26a-5p mimics effectively reduced cell apoptosis of BV-2 cells submitted to OGD/R treatment, and also reduced infarct volume of mice submitted to MCAO/R treatment. These results indicated the protective effect of exosomal miR-25a-5p in cerebral I/R injury, which might be regarded as a novel therapeutic target for cerebral ischemic stroke.

Here, we found that CDK6 was upregulated in MSCs-derived exosomes of MCAO and OGD model, suggesting the essential role of CDK6 in cerebral I/R injury. Previous studies have reported that CDK6 could serve as a target of miRNAs to participate in neuron injury. MiR-99a overexpression inhibits H_2_O_2_ induced G1/S phase transition along with a significant low level of CDK6 in neuro-2a cells (Tao 2015). MiR-424 protects from permanent focal cerebral ischemia injury in mice through targeting CDK6 to inhibit microglia activation (Zhao 2013). However, the regulatory network of CDK6 involved in exosomal miRNAs has not been well studied. To explore the specific mechanism of miR-26a-5p in cerebral I/R injury, bioinformatics prediction was performed and suggested that CDK6 might a direct target of exosomal miR-26a-5p. Then luciferase reporter assay further confirmed the correlation between exosome miR-26a-5p and CDK6. Moreover, MSCs-Exo-miR-26a-5p mimics obviously reduced CDK6 level in BV-2 cells after OGD/R treatment, and also reduced CDK6 level in brain tissues of mice induced by MCAO/R. All these data suggested that the protective effect of MSCs-Exo-miR-26a-5p mimics on cerebral I/R injury both in vitro and in vivo might be mediated by CDK6, providing a new highlight of exosomal miRNAs and cell proliferation-related proteins involved in cerebral I/R injury. Our study suggested that MSCs-derived exosomes overexpressing miR-26a-5p might be applied for the personalized treatment against cerebral ischemic stroke.

However, there was a limitation existing in the study: whether high level of CDK6 reversed the protective effect of MSCs-Exo-miR-26a-5p mimics on cerebral I/R injury, which needed to be further investigated in the subsequent experiments.

## Conclusion

In summary, our results demonstrated that MSCs‑derived exosomes could effectively attenuate I/R injury in vivo and inhibit microglia apoptosis in vitro might through exosomal miR-26a-5p mediated downregulation of CDK6, suggesting that miR-26a-5p might be a novel therapeutic target for cerebral I/R injury.

## Data Availability

Any additional information related to this study is available from the author for correspondence upon reasonable request.
